# Application of *in vivo* imaging techniques to monitor therapeutic efficiency of PLX4720 in an experimental model of microsatellite instable colorectal cancer

**DOI:** 10.18632/oncotarget.19263

**Published:** 2017-07-15

**Authors:** Sarah Rohde, Tobias Lindner, Stefan Polei, Jan Stenzel, Luise Borufka, Sophie Achilles, Eric Hartmann, Falko Lange, Claudia Maletzki, Michael Linnebacher, Änne Glass, Sarah Marie Schwarzenböck, Jens Kurth, Alexander Hohn, Brigitte Vollmar, Bernd Joachim Krause, Robert Jaster

**Affiliations:** ^1^ Department of Medicine II, Division of Gastroenterology, Rostock University Medical Center, Rostock, Germany; ^2^ Core Facility Multimodal Small Animal Imaging, Rostock University Medical Center, Rostock, Germany; ^3^ Oscar-Langendorff-Institute of Physiology, Rostock University Medical Center, Rostock, Germany; ^4^ Molecular Oncology and Immunotherapy, Department of General Surgery, Rostock University Medical Center, Rostock, Germany; ^5^ Institute for Biostatistics and Informatics in Medicine and Ageing Research, Rostock University Medical Center, Rostock, Germany; ^6^ Department of Nuclear Medicine, Rostock University Medical Center, Rostock, Germany; ^7^ Institute of Experimental Surgery, Rostock University Medical Center, Rostock, Germany

**Keywords:** colorectal cancer, mouse model, PLX4720, 5’-fluorouracil, in vivo imaging

## Abstract

**Objectives:**

Patient-derived tumor cell lines are a powerful tool to analyze the sensitivity of individual tumors to specific therapies in mice. An essential prerequisite for such an approach are reliable quantitative techniques to monitor tumor progression *in vivo*.

**Methods:**

We have employed HROC24 cells, grown heterotopically in NMRI Foxn1^nu^ mice, as a model of microsatellite instable colorectal cancer to investigate the therapeutic efficiencies of 5’-fluorouracil (5’-FU) and the mutant BRAF inhibitor PLX4720, a vemurafenib analogue, by three independent methods: external measurement by caliper, magnetic resonance imaging (MRI) and positron emission tomography/computed tomography (PET/CT) with 2-deoxy-2-(^18^F)fluoro-D-glucose (^18^F-FDG).

**Results:**

Repeated measure ANOVA by a *general linear model* revealed that time-dependent changes of anatomic tumor volumes measured by MRI differed significantly from those of anatomic volumes assessed by caliper and metabolic volumes determined by PET/CT. Over the investigation period of three weeks, neither 5’-FU, PLX4720 nor a combination of both drugs affected the tumor volumes. Also, there was no drug effect on the apparent diffusion constant (ADC) value as detected by MRI. Interestingly, however, PET/CT imaging showed that PLX4720-containing therapies transiently reduced the standardized uptake value (SUV), indicating a temporary response to treatment.

**Conclusions:**

5’-FU and PLX4720 were largely ineffective with respect to HROC24 tumor growth. Tumoral uptake of ^18^F-FDG, as expressed by the SUV, proved as a sensitive indicator of small therapeutic effects. Metabolic imaging by ^18^F-FDG PET/CT is a suitable approach to detect effects of tumor-directed therapies early and even in the absence of morphological changes.

## INTRODUCTION

In 2016, colorectal carcinoma (CRC) represented the third most common cancer in females and males and the second leading cause of cancer-related deaths in the United States [1]. While early detection of CRC is associated with an excellent prognosis, there is a strong need for improved therapies for locally advanced and metastatic stages of the disease. A key aspect in this regard is the development of personalized treatment strategies for individual patients that take into account (1) the molecular heterogeneity of the disease and (2) the rapidly growing number of targeted therapeutics and options for tumor-directed immune therapies [2].

Patient-derived tumor cell lines and xenografts provide a versatile tool to study the individual tumor biology and to analyze, understand and potentially predict sensitivity and resistance of tumors to specific therapies [3–6]. A common approach to mimic *in vivo* conditions takes advantage of immunodeficient mice to establish tumors in a heterotopic or orthotopic position, followed by the evaluation of therapeutic approaches. A critical factor for the success of such an approach is the availability of reliable quantitative techniques to monitor tumor progression or regression. While histological investigations are largely restricted to end-point analyses, external measurements with calipers (if applicable) are quite inaccurate and do not provide information about the internal structure of the tumor. Therefore, there is a special need for molecular imaging techniques that enable repeated investigations in living animals [7]. Two essential technologies in this field are small animal magnetic resonance imaging (MRI) and positron emission tomography/computed tomography (PET/CT). Major advantages of MRI are the high resolution and an excellent tissue contrast [7]. MRI not only ensures a precise assessment of tumor size and localization, but, through the measurement of apparent diffusion coefficient (ADC) values, also provides insights into the biological structure of tumor tissue during tumor progression [8]. PET, on the other hand, is widely recognized as a key technology to visualize, with high sensitivity, distinct molecular target structures of a tumor. Integrated PET/CT provides the additional advantage of co-registered molecular and anatomic images, thereby compensating for the relatively poor spatial resolution of PET alone [9]. One of the most commonly used radiopharmaceuticals, 2-deoxy-2-(^18^F)fluoro-D-glucose (^18^F-FDG), is a marker for the uptake of glucose, which is an important parameter for tumor tissue metabolism [10].

In this study, we have employed small animal multiparametric MRI and ^18^F-FDG PET/CT along with external caliper measurements and end-point analyses by histopathology to evaluate an experimental therapy of human CRC in mice. Specifically, we were interested in the consistency of the different types of data and the sensitivity of the relevant methods with respect to an early detection of drug effects. As experimental model, we used HROC24 cells, a patient-derived CRC cell line of low passage number that belongs to the CRC subgroup of microsatellite instable (MSI) tumors [3]. Microsatellite instability (observed in roughly 13 % of all CRCs) is caused by defective DNA mismatch repair and represents the fundamental molecular basis of sporadic hypermutated CRCs and hereditary non-polyposis colorectal cancer syndrome (Lynch syndrome; also known as HNPCC) [11–13]. In a previous *in vitro* study, we could show that HROC24 cells, which express oncogenic *BRAF* (V600E), are highly sensitive to the growth-inhibitory effects of the mutant BRAF inhibitor vemurafenib [4]. We considered this finding of worth to be followed-up since previous experimental studies, which *did not* specifically focus on the subgroup of MSI CRCs, had suggested that vemurafenib is apparently much less efficient in *BRAF* mutant CRC than in malignant melanoma [14].

Recently, a phase II study revealed that single-agent vemurafenib did not show meaningful clinical efficacy in patients with *BRAF* V600E mutant colorectal cancer [15]. As part of combination therapies, however, the concept of targeting the *BRAF* pathway remains viable [2, 15]. To challenge this strategy, we compared the efficiency of monotherapies with the vemurafenib analogue PLX4720 and the routinely used cytostatic drug 5’-fluorouracil (5’-FU) [16], respectively, to the efficiency of a combination of both drugs.

## RESULTS

### Characterization of the heterotopic tumor model

Upon injection into the flanks of NMRI Foxn1^nu^ mice, HROC24 cells formed macroscopic tumors within less than two weeks. Subsequently, the mice were randomized into four experimental groups and treated for three weeks with PLX4720, 5’-FU, both drugs, or vehicle only. All mice of the control cohort and all individuals except of one of each treatment group survived throughout the course of the study. Further details are outlined in the “materials and methods” section.

**Figure 1:**
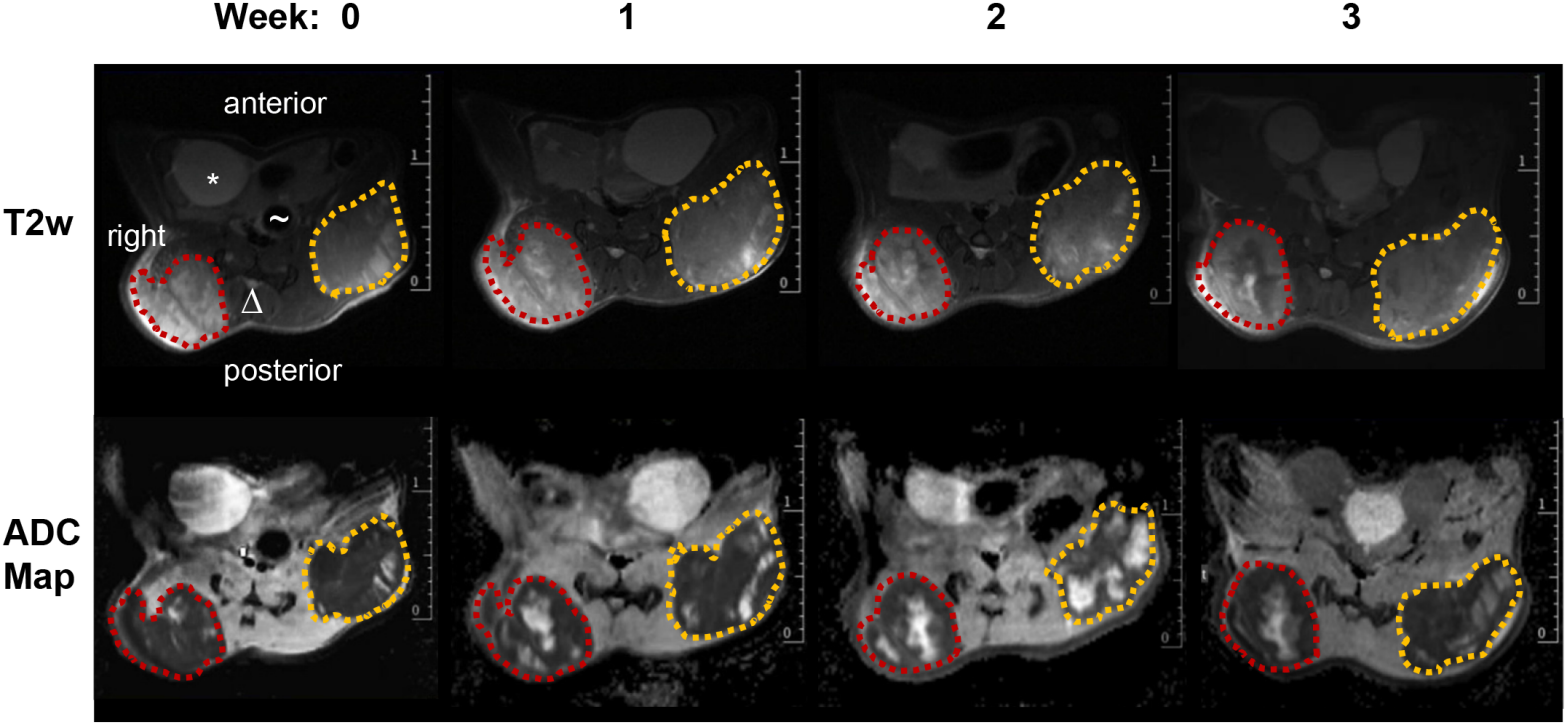
MR images of a mouse from the control group top row: representative T2 weighted transversal images (red dotted line: right tumor volume; orange dotted line: left tumor volume, * bladder, Δ spine, ∼ gut); bottom row: ADC-maps derived from DWI for four different time points (0, 1, 2 and 3 weeks) showing heterogeneous tumors with solid tumor mass (dark areas represent tissue with low ADC values) and pervading necrotic/cystic areas (bright areas with high ADC values).

**Figure 2:**
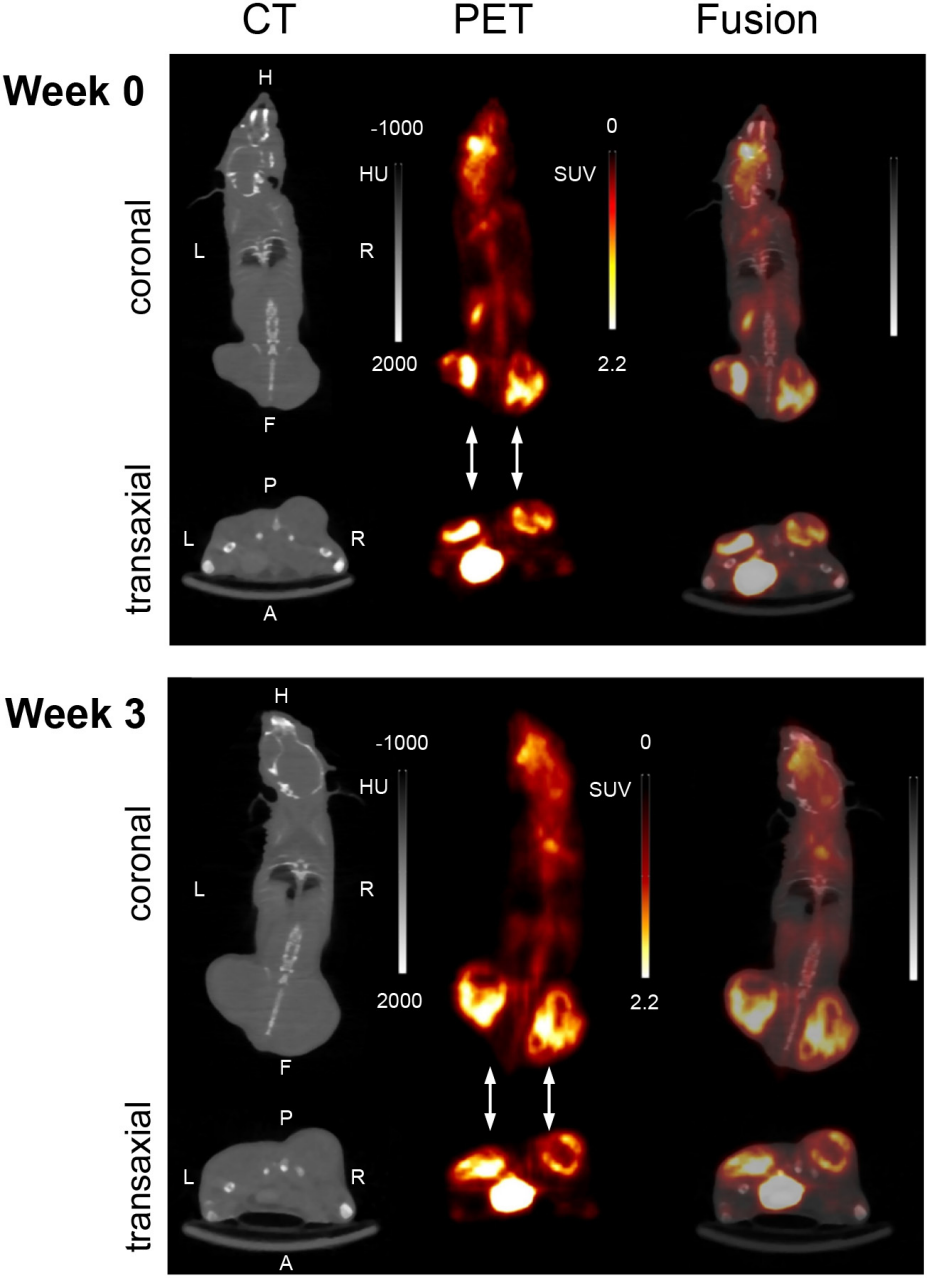
Representative PET/CT images (summed images; coronal and transaxial slices) at two experimental time points (week 0, week 3) The images were derived from the same mouse as in Figure 1: A 15 min imaging scan was performed 60 min after intravenous injection of ^18^F-FDG. The flank tumors are pointed out by arrows. HU; Hounsfield units; SUV; standardized uptake value.

**Figure 3:**
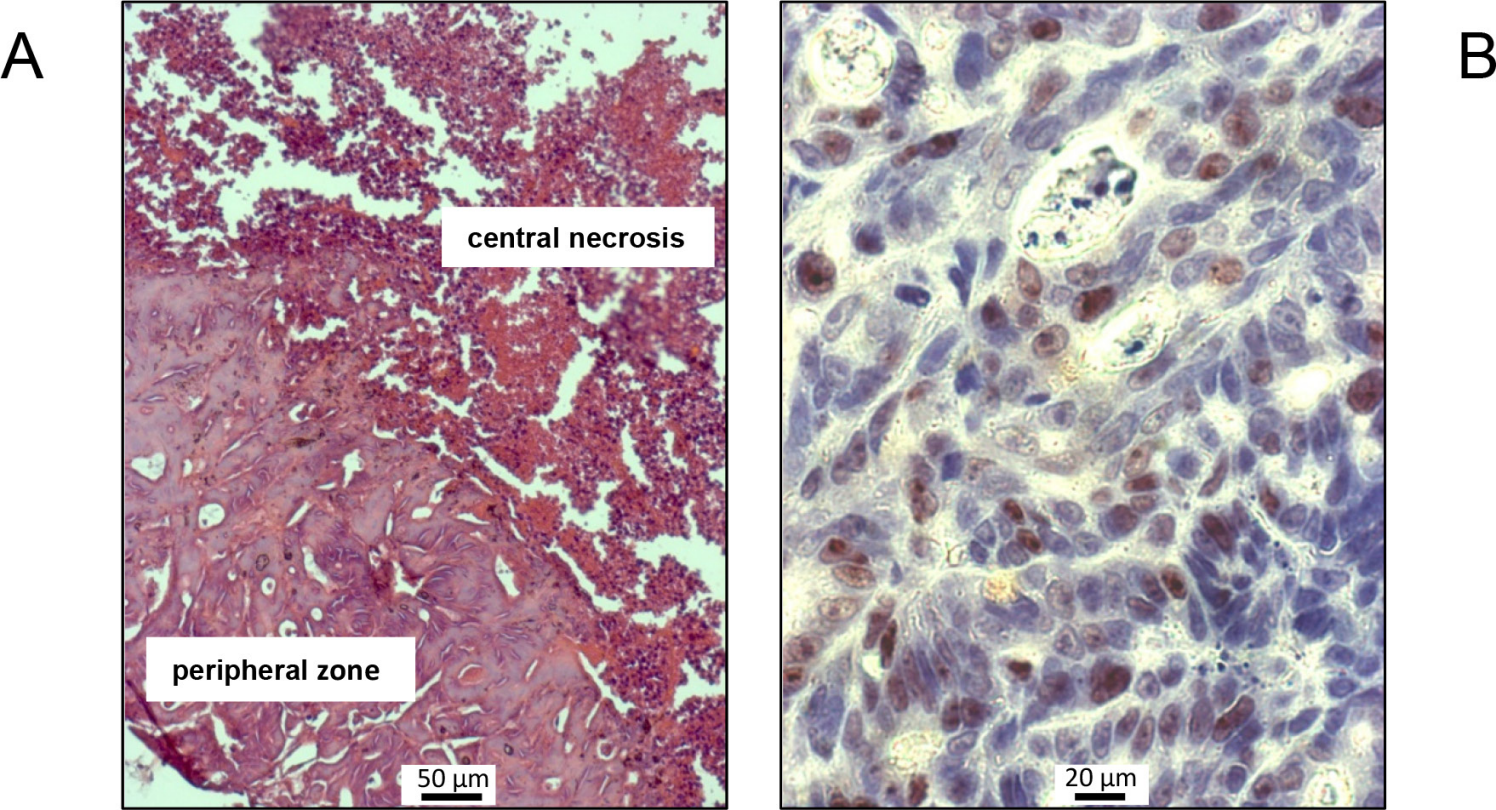
Representative H&E **(A)** and Ki-67 **(B)** stains of tumor tissue, exemplarily shown for a mouse of the control group. **(A)** Viable colorectal cancer tissue in the peripheral region surrounding a central necrosis. **(B)** Ki-67-positive cells with brown-stained nuclei are present in large numbers.

**Figure 4:**
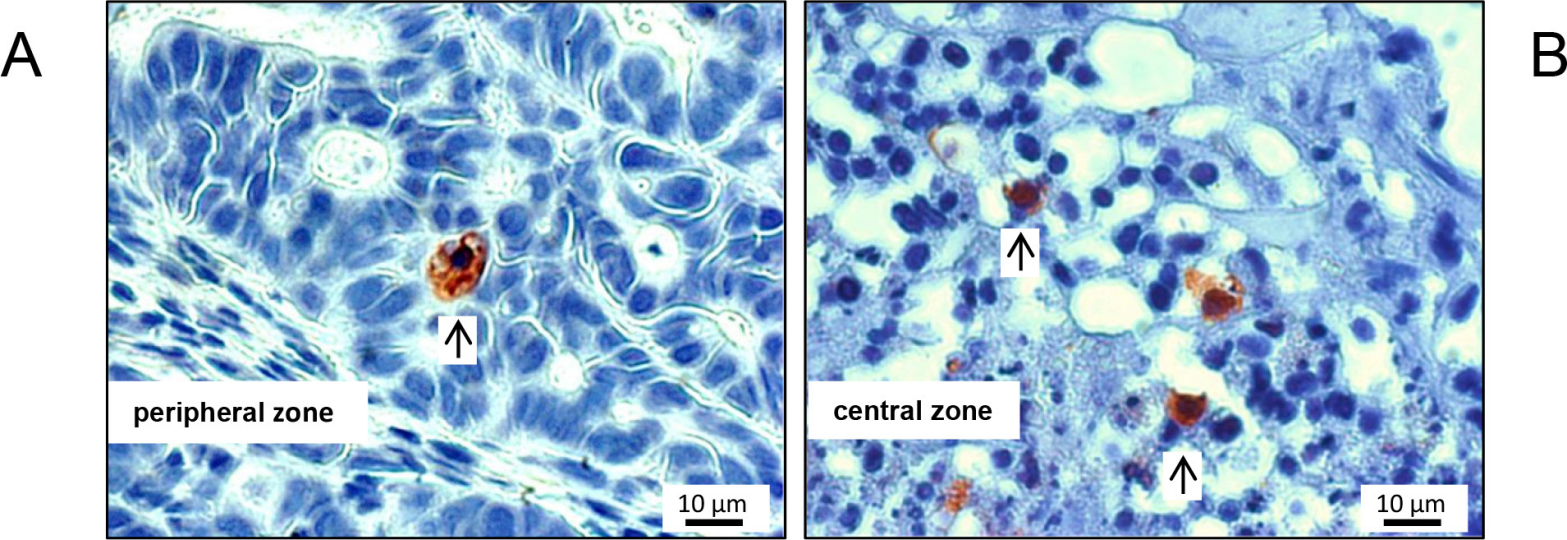
Typical M30 CytoDEATH stains of apoptotic cells, exemplarily shown for a mouse of the control cohort Apoptotic cells were very scarce in the periperal zone of the tumors **(A)**, and somewhat more frequent (but still rare) in the transition zone to the necrotic centre of the xenografts **(B)**. Arrows point to positive-stained cells.

Over the entire period of investigation, the tumors were accessible to external measurement by caliper and clearly detectable both by MRI and PET/CT imaging with ^18^F-FDG (Figure 1 and 2). Both imaging techniques indicated a heterogeneous structure of the tumor with vital tumor tissue in the peripheral zone and central necrotic areas. Haematoxylin and eosin (H&E) staining of the tumor tissue at the time of necropsy confirmed these findings (Figure 3A). Cells expressing the proliferation marker Ki-67 [17] (Figure 3B) were exclusively found in the peripheral regions of the tumors. Apoptotic cells were in general very scarce and almost exclusively found in the transition zone between vital and necrotic areas (Figure 4).

### Assessment of anatomic and metabolic tumor volumes

**Figure 5:**
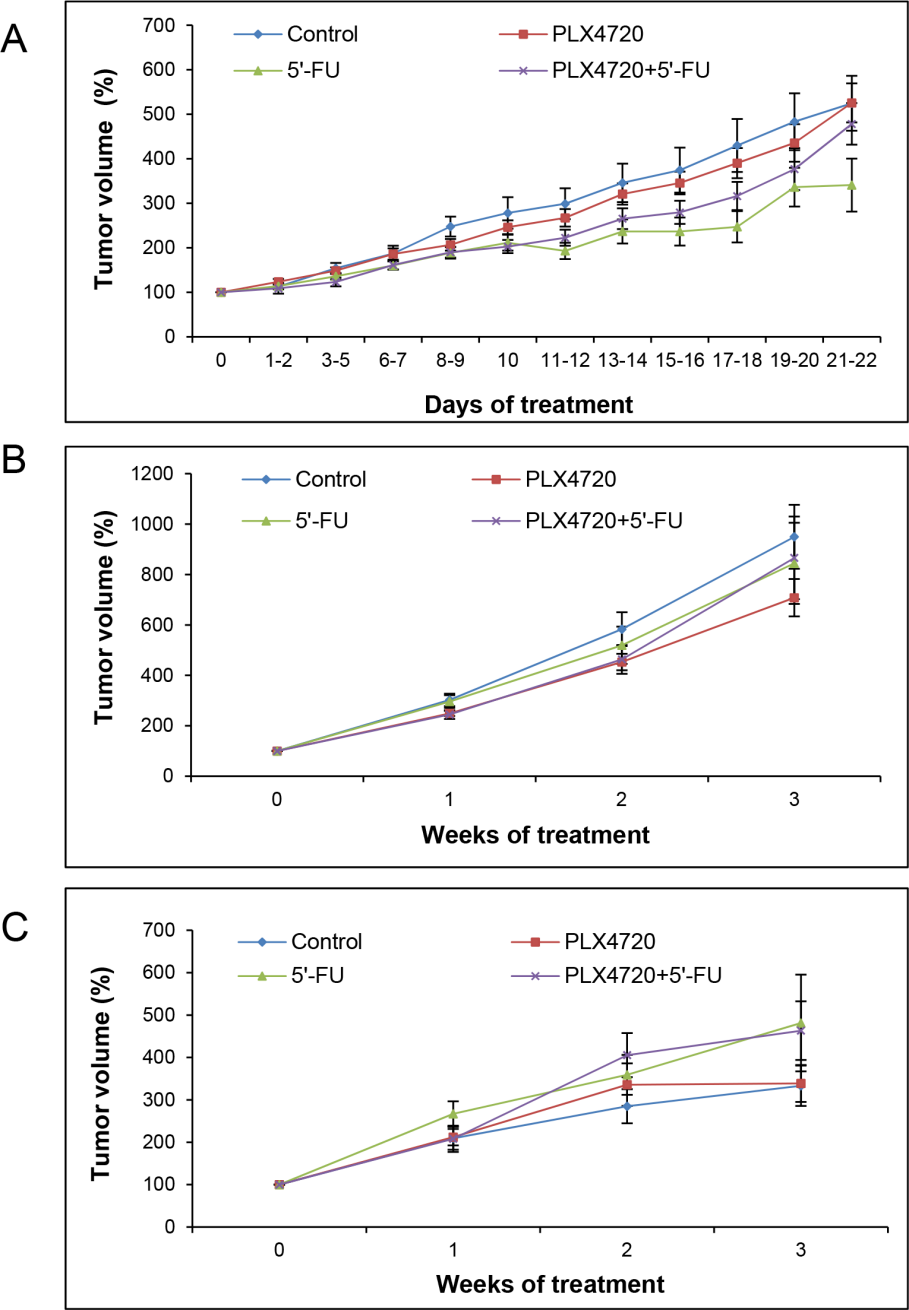
Assessment of anatomic and metabolic tumor volumes Mice carrying two flank tumors of HROC24 cells were treated with PLX4720, 5’-FU, a combination of both drugs, or served as controls (n = 8-9 mice per experimental group). At the indicated time points, anatomic tumor volumes were assessed employing a caliper **(A)** and measured by MRI **(B)**, whereas metabolic tumor volumes were determined by PET/CT **(C)**. One hundred percent tumor volume corresponds to the tumor size prior to the start of treatment. Data are presented as mean ± SEM (n = 12-18 samples per time point and method; variables: number of tumors per mouse that fulfilled the inclusion criteria, survival time of the animals and availability of evaluable data). There were no statistically significant differences between the experimental groups.

Anatomic tumor volumes assessed by caliper and MRI as well as metabolic volumes determined by PET/CT imaging (Figure 5A-5C) were subjected to analysis of variance by means of *general linear model-repeated measures* (GLM-RP). Here and in all related analyses, there was no significant influence of tumor location (left or right hind flank) so that for exploratory research all tumors of mice with the same treatment regime could be summarized in one group.

As expected, there was a highly significant effect of the factor “time” in a way that the tumor volumes increased over the period of investigation (p<0.001 for any time points). In contrast, however, no significant difference could be detected between the four experimental groups (p=0.564), suggesting a lack of therapeutic effects.

**Figure 6:**
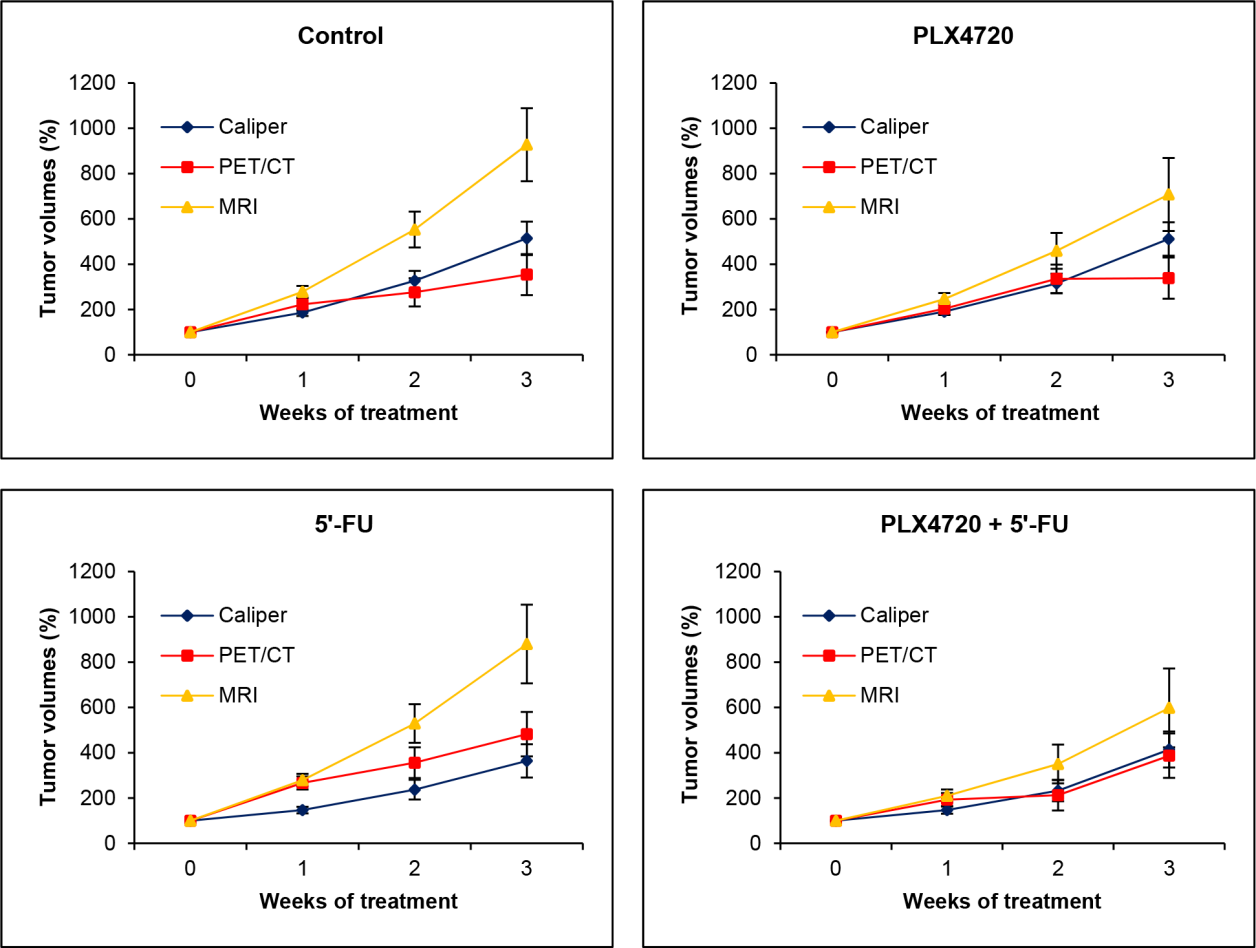
Comparison of volumetric data obtained by caliper, MRI and PET/CT The data presented in Figure 5 were used to calculate (by GLM-RP model) estimated marginal means ± SEM of anatomic and metabolic volumes over time for each of the four experimental groups. A value of one hundred percent corresponds to tumor volumes prior to treatment (week 0).

Interestingly, further analyses revealed significant differences between the (larger) changes of anatomic volumes determined by MRI on one hand and the (smaller) changes of anatomic volumes assessed by caliper as well as metabolic volumes determined by PET/CT imaging on the other hand (p<0.03 each). The differences are illustrated in Figure 6 for all experimental groups over time. In the case of the metabolic volumes, this finding is likely to be explained by the fact that intratumoral regions without uptake (central necrosis) were not considered for volume calculation. Measurements by caliper might lead to a misjudgement of tumor growth due to an inadequate consideration of the third dimension (tumor depth).

### Apparent diffusion constant (ADC) and standardized uptake value (SUV)

**Figure 7:**
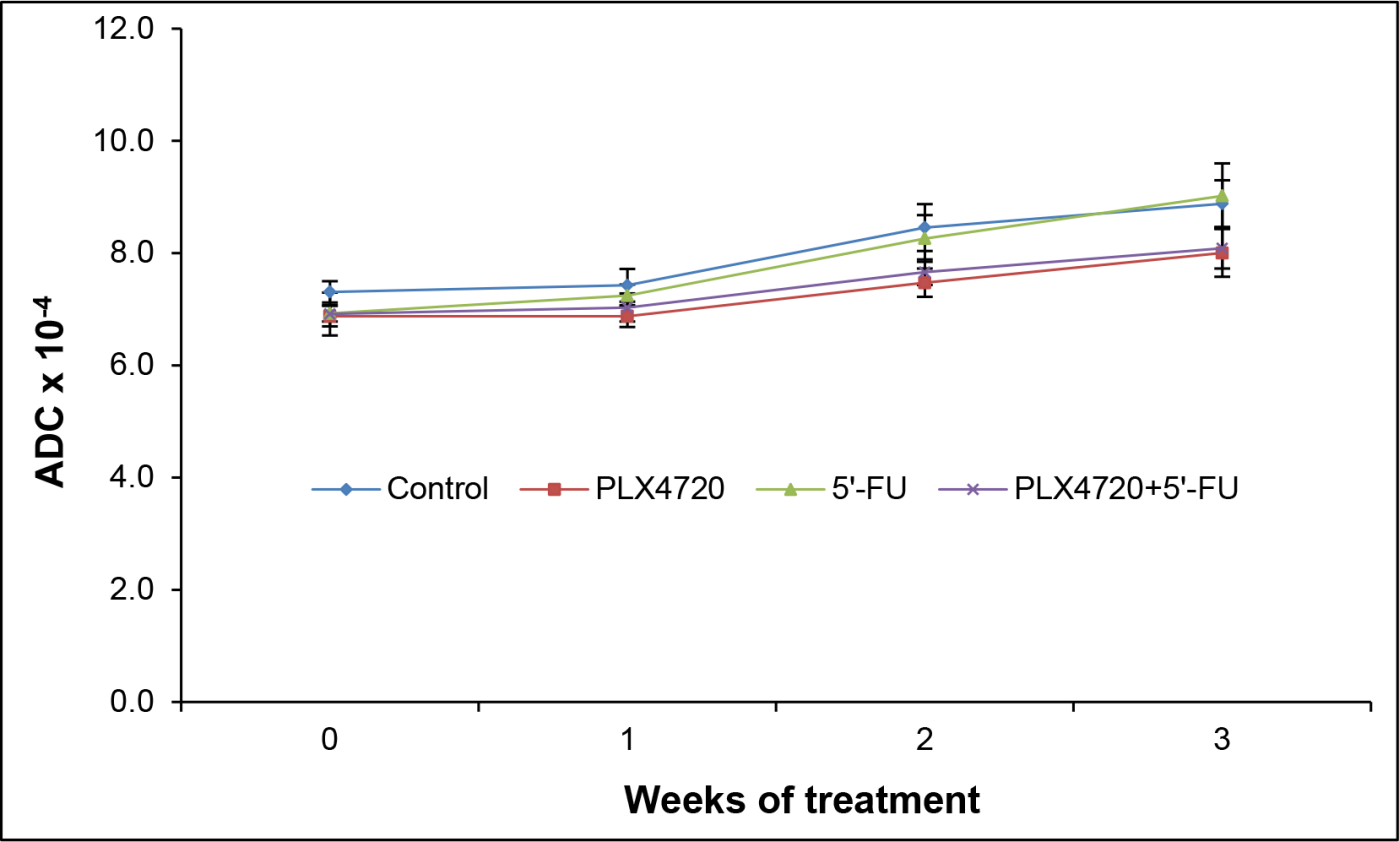
ADC values of the tumors At the indicated time points of treatment, mice of the four experimental groups (n = 8-9 per treatment protocol) were subjected to MRI, and ADC values of the tumors were determined. Data are presented as mean ± SEM (n = 13-18 samples per time point; variables as described in Figure 5). There were no statistically significant differences between the experimental groups.

ADC values are indicators of the magnitude of diffusion of water molecules within the tissue. Decreases of the cellular density are associated with increased ADC values and may therefore suggest a therapeutic response [18]. In this investigation, no significant changes of the ADC over time in the context of treatment were observed (Figure 7).

**Figure 8:**
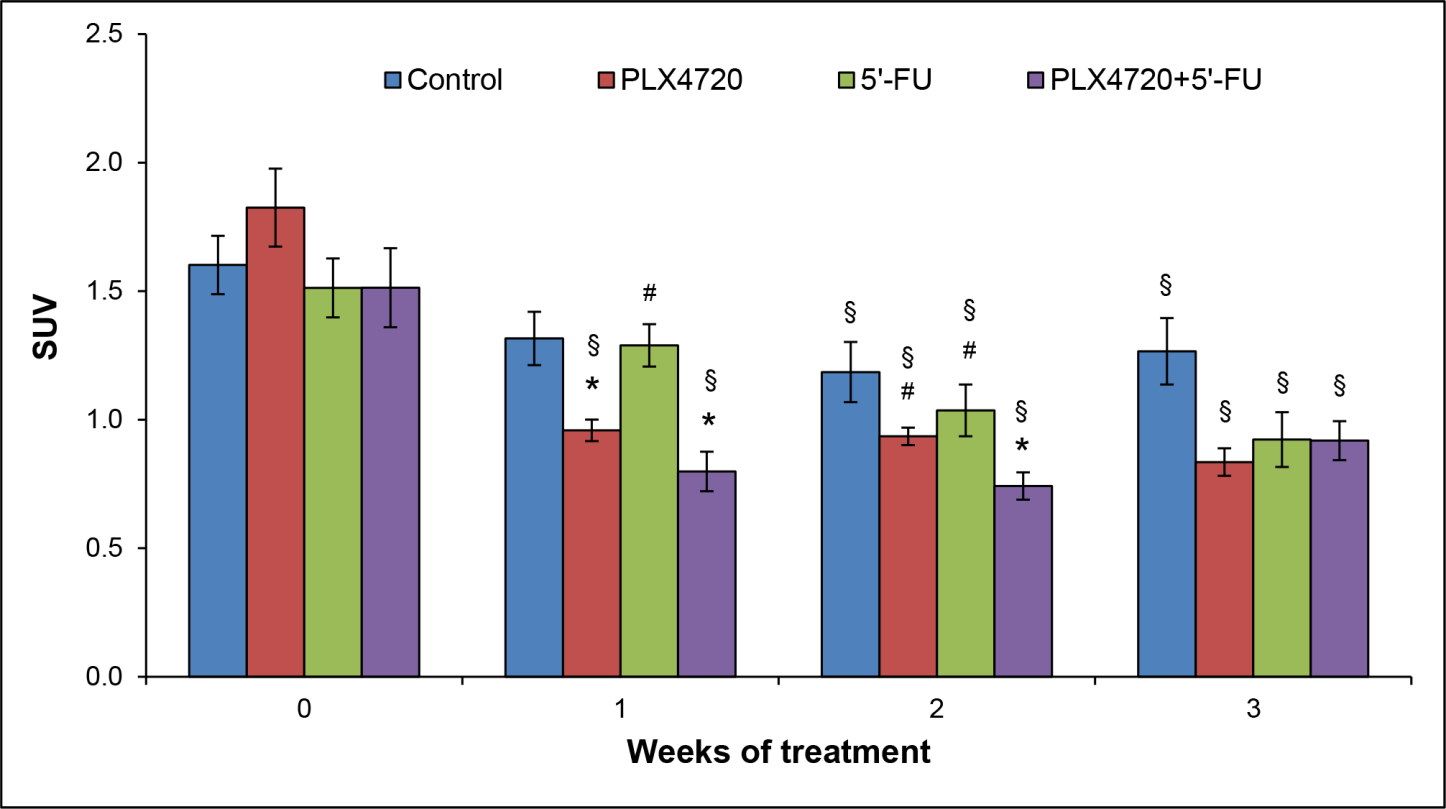
SUV analysis Prior to the initiation of treatment and 1-3 weeks thereafter, mice of the four experimental groups (n = 8-9 per treatment protocol) were subjected to PET/CT, and mean SUVs of the tumors ± SEM were determined (n=12-17 samples per time point; variables as described in Figure 5). ^§^ p<0.05 vs. week 0 (Friedman test followed by Wilcoxon tests). * p<0.05 vs. controls and ^#^ p<0.05 vs. combination of PLX4720 + 5’-FU (same time point; Kruskal-Wallis test followed by Mann-Whitney U tests).

SUVs were expressed as mean values and used as a parameter of normalized intratumoral radioactivity concentrations [19]. In contrast to detections of anatomic/metabolic volumes and ADC values, measurements of mean SUV of tumors revealed differences between the experimental groups (Figure 8): For all three cohorts with specific therapies, tumoral mean SUVs were significantly lower after completion of treatment (week 3) than before its initiation (week 0). PLX4720-containing therapies reduced the average SUV of tumors also at earlier time points (weeks 1 and 2). For the control group, a diminished SUV was observed in week 2 only. Moreover, the comparison of treatments at individual time points indicated that application of PLX4720 (alone or combined with 5’-FU) was associated with a significantly lower SUV in week 1 and week 2.

**Figure 9:**
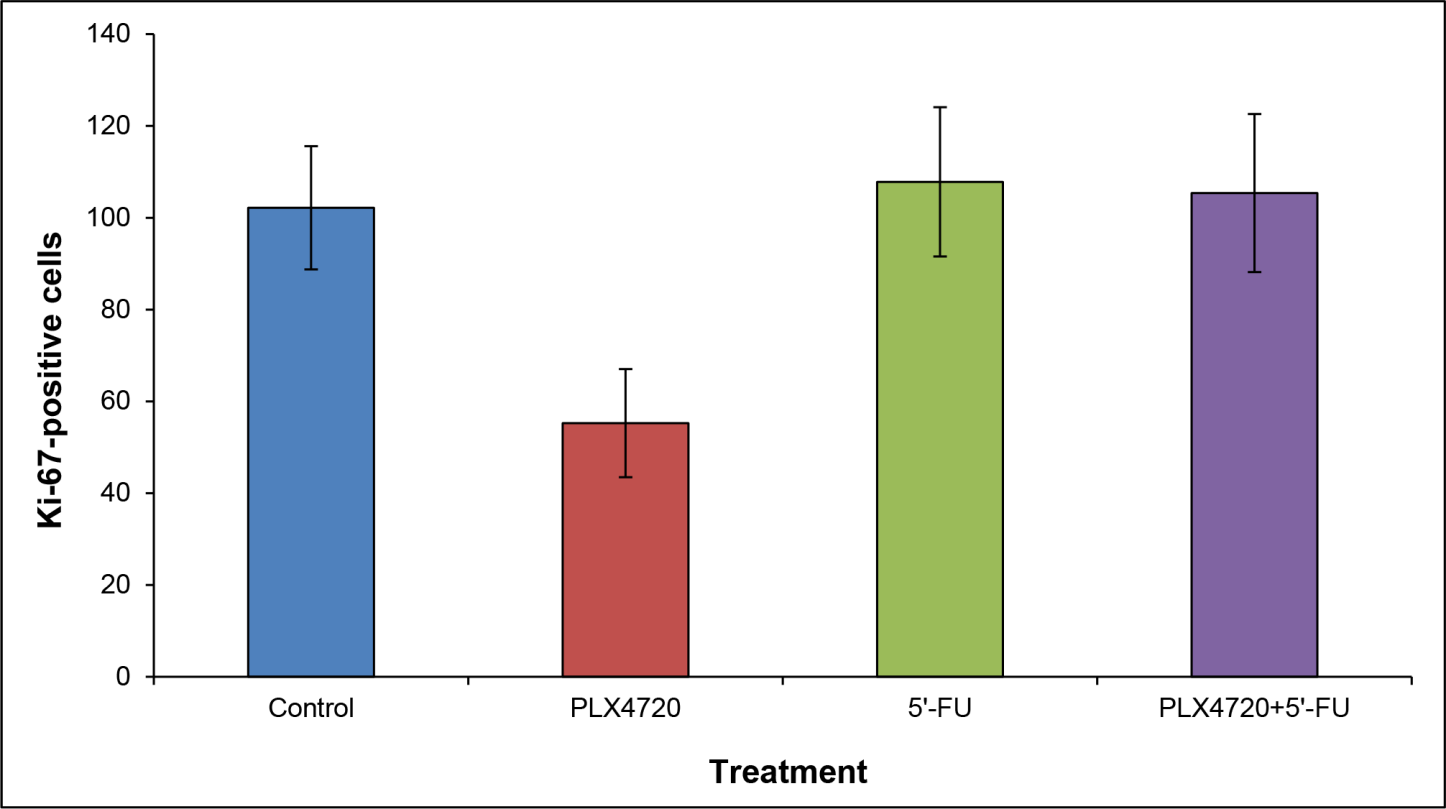
Quantification of Ki-67-positive cells Staining and cell counting were performed as described in the “materials and methods” section. Data are presented as numbers of Ki-67-positive cells out of 300 tumor cells (mean ± SEM; n = 15-17 samples). There were no statistically significant differences between the experimental groups.

Together, these ^18^F-FDG PET/CT findings point to a therapeutic effect of PLX4720-containing therapies. In line with the transient nature of this effect, histological evaluation at the time of necropsy revealed no differences with respect to tumor morphology and the presence of apoptotic cells between the experimental groups (data not shown). Furthermore, variations of the number of Ki-67-positive cells among the groups were statistically not significant (Figure 9).

## DISCUSSION

Inhibition of mutant BRAF by specific kinase inhibitors has proven a successful strategy in the treatment of malignant melanoma [20, 21]. Although oncogenic *BRAF* mutations are also present in a subgroup of CRC patients, these patients do not benefit from mutant BRAF-directed therapies to date [15]. MSI tumors form a subclass of CRCs that is characterized by DNA repair defects [11–13]. MSI is associated with a better prognosis of CRC patients [22] and has also been linked to alterations of chemosensitivity. Specifically, the benefit of an adjuvant treatment with 5’-FU, the most widely used chemotherapeutic agent in advanced stage CRC patients, is a matter of ongoing discussion [23]. How the MSI status affects the response of CRC patients to targeted therapeutics, such as mutant BRAF inhibitors, has not been fully evaluated yet.

We have previously shown that low-passage HROC24 cells, established from a microsatellite instable CRC, are highly sensitive to PLX4720 *in vitro* [4]. The observation prompted us to ask if these findings can be reproduced in a preclinical setting employing NMRI Foxn1^nu^ mice. We therefore took advantage of traditional and advanced technologies to monitor tumor progression *in vivo*. Accordingly, we considered the systematic comparison of the corresponding findings as an equally important aim of this study. The results of volumetric measurements by caliper, MRI and PET/CT with ^18^F-FDG consistently showed that neither PLX4720 and 5’-FU alone nor the combination of both drugs displayed a significant effect on the growth of HROC24 tumors over the investigation period of three weeks. These negative findings are supported by the results of ADC measurements and end-point analyses (histological evaluation; Ki-67-staining), which did also not indicate differences between the experimental groups. Interestingly, however, measurements of mean SUVs of the tumors revealed *transient* therapeutic effects of PLX4720-containing therapies (with and without 5’-FU). Since these effects were missed by the volumetric measurements, we consider the detection of normalized intratumoral radioactivity concentrations as the most sensitive approach, and therefore PET/CT with ^18^F-FDG as the method of choice for the detection of small/transient therapeutic effects in the context of this study. Noteworthy, metabolic imaging by ^18^F-FDG PET/CT proved suitable to detect effects of tumor-directed therapies early in the course of treatment and even in the absence of morphological changes. The significance of ^18^F-FDG PET/CT data, especially SUV measurements, for the preclinical evaluation of antitumor drugs should therefore be further elucidated in follow-up studies.

There are limitations of our investigations that need to be mentioned. Thus, heterotopic mouse models provide only limited insights into the biology of human CRC. Still, on the road towards a personalized treatment of CRC they may represent an important bridge between mere *in vitro* studies and clinical trials. Furthermore, application of PLX4720 via chow, although representing an established procedure, does not allow for an effective control of dosage and might result in an inefficient drug concentration in the tumor tissue. Last but not least, restrictions of the animal model (minimum and maximum tumor sizes) limited the duration of therapy to three weeks, leaving the possibility of enhanced or delayed drug effects after longer periods of treatment.

As mentioned above, the effectiveness of 5’-FU in microsatellite instable CRC is under debate. Specifically, a systematic review revealed no statistically significant effect of 5’-FU for microsatellite high (MSI-H) patients for disease-free survival or overall survival. These findings were in sharp contrast to the results for MSI-stable CRC, where the drug significantly improved survival rates [23]. The complete failure of 5’-FU, at the established dose of 20 mg/kg [24], to inhibit growth of HROC24 tumors was nevertheless unexpected and remains to be addressed in future experiments.

In summary, we suggest SUV, determined by PET/CT with ^18^F-FDG, as a sensitive parameter to monitor drug responsiveness in a preclinical model of CRC. Measurements of anatomic volumes by MRI and metabolic volumes by PET/CT provide non-redundant information and may complement each other in a meaningful manner. The difference between changes of anatomic volumes determined by MRI and by caliper points to a systematic problem of the latter method, which does not accurately consider the tumor depth. Although 5’-FU and PLX4720 displayed very limited effects in this investigation, the *concept* of mutant BRAF inhibition in CRC with MSI should be challenged in follow-up studies with additional MSI cell lines and further inhibitors of the BRAF pathway.

## MATERIALS AND METHODS

### Tumor model

The MSI cell line HROC24 was established from a primary resection specimen of a 98 years old male CRC patient and grown in culture as described before [3, 4]. HROC24 cells are mutant for *BRAF* (V600E) and *APC* but wild-type for *TP53* and *KRAS* [3].

NMRI Foxn1^nu^ mice were bred in the animal facility of the Rostock University Medical Center and maintained in specified pathogen-free conditions. All experiments were performed according to the guidelines of the local animal use and care committee, which also approved this study (Landesamt für Landwirtschaft, Lebensmittelsicherheit und Fischerei Mecklenburg-Vorpommern, permit number for the study: LALLF M-V/TSD/7221.3-1-033/14). The mice had access to water and standard laboratory chow (as specified below) ad libitum. All animals received humane care according to the German legislation on protection of animals and the Guide for the Care and Use of Laboratory Animals (NIH publication 86–23, revised 1985), and all efforts were made to minimize suffering. Six to eight-weeks-old female NMRI Foxn1^nu^ mice were injected subcutaneously into the left and right hind flank with 5×10^6^ HROC24 cells. Over the entire period of investigation, tumor growth was monitored at least three times per week by external measurement using a caliper, and volumes of outgrowing tumors were evaluated according to the formula: width^2^ × length × 0.52 [25, 26].

On days 11-12 after cell injections, mice were randomized into four experimental groups of 8-9 individuals and treatment was initiated: (1) Control mice were fed a standard rodent chow diet (Broogarden, Lynge, Denmark). The mice also received intraperitoneal injections of phosphate-buffered saline twice a week. (2) Animals of the 5’-FU group were injected twice a week at a dose of 20 mg/kg and fed the standard rodent chow diet. (3) A third group of mice obtained PLX4720 (Plexxikon Inc., Berkeley, California, USA) provided through ad libitum chow at a dose of 417 mg/kg (Broogarden). (4) Mice of the fourth group received a combined treatment with 5’-FU and PLX4720 as described above. After 21 days of treatment, mice were sacrificed by an overdose of ketamine/xylazin hydrochloride and tumors were harvested for further analysis.

One mouse of the PLX4720 group had to be euthanized on day 15 of treatment before the tumor burden became intolerable. Furthermore, one mouse of the 5’-FU group and one mouse of the PLX4720 + 5’-FU group had to be sacrificed on days 10 and 17, respectively, because of progressive weight loss. In all these cases, the data of all available time points were included into further analyses. In contrast, tumors of less than 5 mm in length at all time points (including day 0) were disregarded.

### Animal PET/CT imaging

Small animal PET/CT imaging with ^18^F-FDG was performed 1-2 days prior to the initiation of treatment and repeated on days 6-7, 13-14 and 20-21 of therapy (four investigations per mouse in total). For all scans, animals were anaesthetized by isoflurane (1.5–2.5 %) supplemented with oxygen during preparations and imaging sessions. Mice received a mean dose of 17.50 ± 1.80 MBq ^18^F-FDG intravenously via a microcatheter placed in a tail vein. After an uptake period of 60 min, mice were imaged in prone position in a preclinical PET/CT scanner (Inveon PET/CT, Siemens Medical Solutions, Knoxville, TN, USA) for 15 min. The animals were kept at constant temperature of 38°C by an electrical heating pad. In addition, respiration was monitored during the whole imaging period. For attenuation correction and anatomical references whole body CT scans were acquired. Each PET data set was corrected for random coincidences, dead time, scatter and attenuation. CT images were reconstructed with a Feldkamp algorithm. PET data were first Fourier rebinned into a 2D dataset from which real-space images were reconstructed with an ordered subset expectation maximization (OSEM) algorithm with 16 subsets and 4 iterations. Data were decay-corrected to the time of injection. Metabolic volumes and SUVs were determined using PMOD v3.7 preclinical imaging software (PMOD Technologies, Zurich, Switzerland).

### Morphological and diffusion-weighted 7 Tesla MRI

MRI was performed on anesthetized mice (1.5–2.5 % isoflurane in oxygen) at the same time points as PET/CT (1-2 days before and 6-7, 13-14 and 20-21 days after the initiation of therapy; four investigations per mouse in total). Animal respiration rate and body temperature were monitored continuously and respiration rate was maintained between 35 and 50 breaths/min.

MR imaging of the mice was performed using a 7 Tesla small animal MRI scanner (BioSpec 70/30, 7.0 T, 440 mT/m gradient strength, Bruker, Ettlingen, Germany) with a 1 H transmit resonator (86 mm Resonator, Bruker, Ettlingen, Germany) and a 2-by-2 receive-only surface coil array (Bruker, Ettlingen, Germany) positioned on the back of the mice. The imaging protocol included morphological T2-weighted turboRARE and diffusion-weighted (DWI) imaging sequences. Tumor size was assessed in high resolution T2-weighted images of transversal plane (repetition time: due to respiratory gating approximately 4.200 ms; echo time: 26.0 ms; field of view: 42 mm × 24 mm; matrix: 351 × 200; voxel size: (0.12 × 0.12 × 0.75) mm^3^; 35 to 50 slices depending on tumor size; acquisition time: 10 min).

The ADC value was calculated by least square monoexponential fit of the pixel-by-pixel signal intensity for the different b-value images of a spin echo DWI-sequence (four b values, b_1_- b_4_:(100, 350, 700, 1.000) s/mm^2^, one b_0_ image and three orthogonal gradient directions; repetition time: 2.000 ms; echo time: 25 ms; field of view: 42 mm × 24 mm; matrix: 192 × 109; 35-50 slices of 0.75 mm per slice in transversal plane; acquisition time: 30-35 min, depending on tumor dimensions and number of slices).

Images and calculated ADC-maps were analyzed employing ITK-Snap software (Penn Image Computing and Science Laboratory “PICSL”, University of Pennsylvania, USA) [27]. The tumor volume and ADC evaluation is based on slice-wise region of interest placement. ADC calculation was performed as described by Jung *et al.* 2012 [28].

### Histology and immunohistochemistry

For histology, tumors were fixed in 4% formaldehyde phosphate buffer overnight and processed for paraffin embedding. Routine H&E staining for assessment of tumor histology was performed on 4 μm sections using standard procedures.

Apoptotic cells were detected employing the M30 CytoDEATH assay (Roche Diagnostics, Mannheim, Germany), which detects the caspase cleavage product of cytokeratin 18. Therefore, deparaffinated tumor sections were stained following the instructions of the manufacturer. Subsequently, the slides were counterstained with Mayer’s hemalaun solution, dehydrated by four short incubations in ethanol and xylene (two times each) and embedded in Pertex (MEDITE, Burgdorf, Germany). For the detection of proliferating cells, expression of Ki-67 was used as a surrogate marker [17]. Therefore, deparaffinated sections of tumor tissue were stained employing anti-Ki-67 antibody (eBioscience, Frankfurt, Germany) and an avidin-biotin-peroxidase (ABC) technique [29], followed by counterstaining with Mayer’s hemalaun solution. Ki-67 positive-stained cells were quantified by evaluating 3 x 100 nuclei, located in three representative areas along the invasive front of the tumor, per section.

### Statistical analyses

All data were processed using IBM SPSS Advanced Statistics 22.0. Values were expressed as mean ± standard error of the mean (SEM) for the indicated number of samples. Anatomic and metabolic tumor volumes (related to time point 0) were analyzed and compared with repeated measure ANOVA for (factor) “*treatment”* and for (within-subjects factors) “*measuring method”*, “*time”* and “*tumor location”* employing GLM-RP. Here, estimated marginal means and their standard errors are given.

For all other data, analysis of variance was performed employing the Kruskal-Wallis test for unpaired samples and the Friedman test for paired data, respectively. If appropriate, subgroups were tested pairwise using the Mann-Whitney U test (unpaired samples) or the Wilcoxon signed rank sum test (paired data), two-sided. Normal distribution of measurements was checked using the Kolmogorov-Smirnov test. P < 0.05 (Bonferroni-adjusted for multiple testing) was considered to be statistically significant.
